# Bilateral rubber hand illusion induced by unilateral visuotactile stimulation

**DOI:** 10.1177/20416695241278277

**Published:** 2024-09-02

**Authors:** Shinya Yamamoto

**Affiliations:** Department of Information Technology and Human Factors, 73459 National Institute of Advanced Industrial Science and Technology (AIST), Tsukuba, Japan; Graduate School of Comprehensive Human Sciences, 13121 University of Tsukuba, Tsukuba, Japan

**Keywords:** bilateral hands, body image, rubber hand illusion, sense of body ownership

## Abstract

The rubber hand illusion involves the sense of body ownership of a fake hand. We showed that concurrent visuotactile stimuli to *unilateral* rubber and real hands can induce the embodiment of *bilateral* rubber hands when both rubber hands are positioned on the table. This phenomenon indicates that the brain has an integrated representation of the sense of body ownership for both hands.

In the rubber hand illusion (RHI), individuals perceive a rubber hand as their own when visual input that the rubber (fake but visible) hand is touched and tactile input that their physical (real but invisible) hand is touched are concurrently presented (Botvinick & Cohen, 1998). This illusion provides insight into the realisation of a sense of body ownership (SoBO) in the brain. If a mannequin's whole body (i.e., trunk, upper and lower limbs) can be viewed from its head, the concurrent visuotactile stimuli to fake and real hands on one side can induce a SoBO for the whole body (Petkova et al., 2011). However, whether a SoBO for two remote parts of a body can be induced by presenting concurrent stimuli to one part of the body remains unknown. Therefore, we examined whether concurrent visuotactile inputs from *unilateral* rubber and real hands induced a SoBO in *bilateral* rubber hands under the condition that only the two rubber hands (not the trunk and legs) were visible.

Seven participants (five men and two women; six right-handed and one left-handed) were included. The experimental setup is illustrated in Figure 1. The right and left rubber hands were positioned on a table in a palms-down position in front of the participants. The participants positioned their hands on the outer sides of the rubber hands with their palms down. The participants’ hands and rubber hands were separated using plastic boards. The participants’ hands, trunk, and proximal ends of the rubber hands were covered with towels to hide them from the participants.

**Figure 1.**
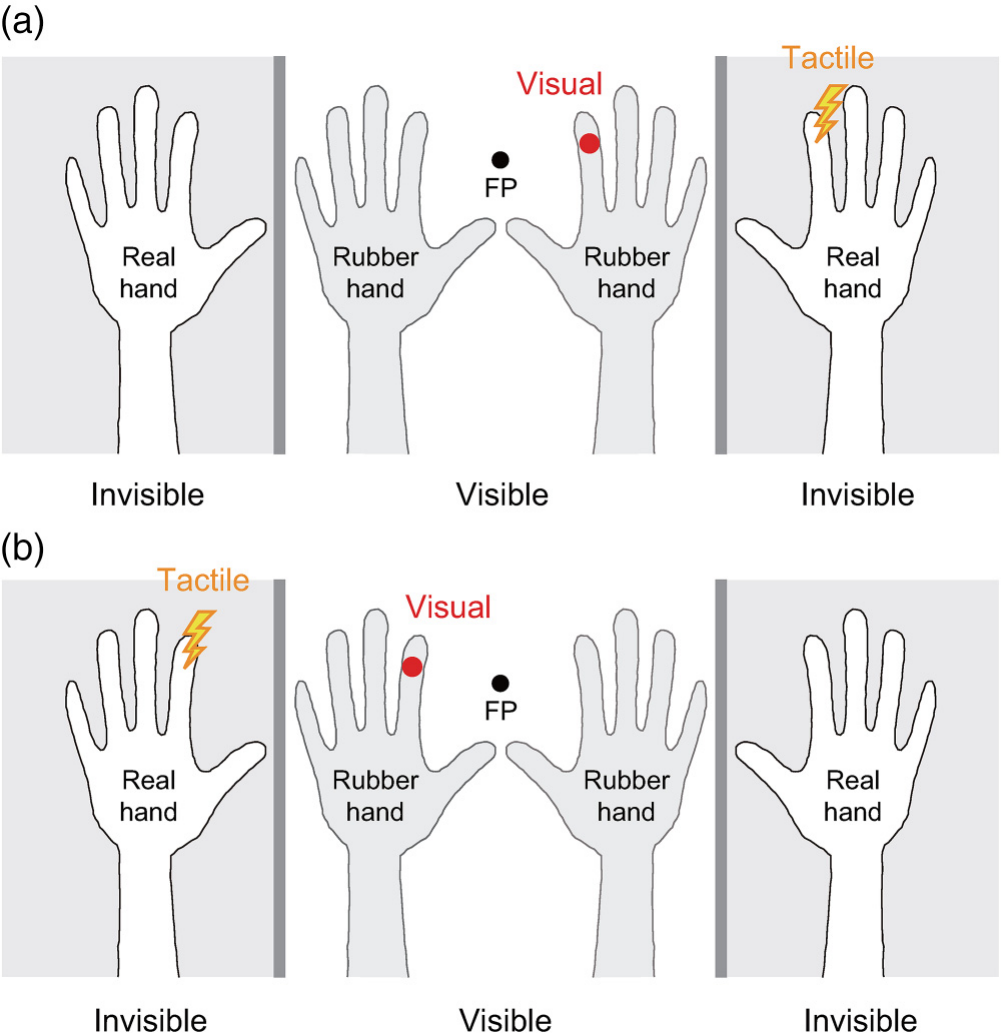
Experimental setup. Bilateral rubber hands and participants’ real hands are positioned on a table. Concurrent visuotactile stimuli are delivered to one side of the rubber and real hands (a: right, b: left). FP indicates a fixation point.

Flashes from light-emitting diodes (LEDs) and electrical current pulses were used as concurrent visuotactile stimuli. A red LED was embedded in the index finger of each rubber hand; the participants could see their flashes through the rubber skin. Electrical current pulses were delivered to the participants’ index fingers using surface electrodes. The current strength was 1.2 times the sensory threshold for each participant. The LED flashes and electrical pulses each had a duration of 10 ms. Concurrent visuotactile stimuli for the right rubber/real hands (Figure 1(a)) or left hands (Figure 1(b)) were presented every 1.2 s for 8 min in each experimental session. Each participant participated in 4–10 sessions. In each session, visuotactile stimuli were delivered to the fake and real hands (e.g., right hands) of one side, while no stimulation was provided to the contralateral fake and real hands (e.g., left hands). Participants were instructed to gaze at a fixation point between the two rubber hands during the experiments.

During the sessions, participants were asked to verbally report their introspection of their SoBO immediately after any changes in their perception occurred. Accordingly, their SoBO for each time point was classified as: [1] SoBO for both hands, [2] SoBO for the hand ipsilateral to the stimulation, [3] SoBO for the hand contralateral to the stimulation, and [4] SoBO for no hands. The average percentages of the durations for each SoBO category for the individual participants are shown in Figure 2(a). The duration of the SoBO for both hands ([1]) was significantly longer than that for the ipsilateral hand ([2], *P *= 0.016, *paired t-test*) and that for the contralateral hand ([3], *P *= 0.012, *paired t-test*). Thus, the duration of the SoBO for both hands ([1]) occupied the majority of that for any SoBO ([1] + [2] + [3]) (Figure 2(b)).

**Figure 2.**
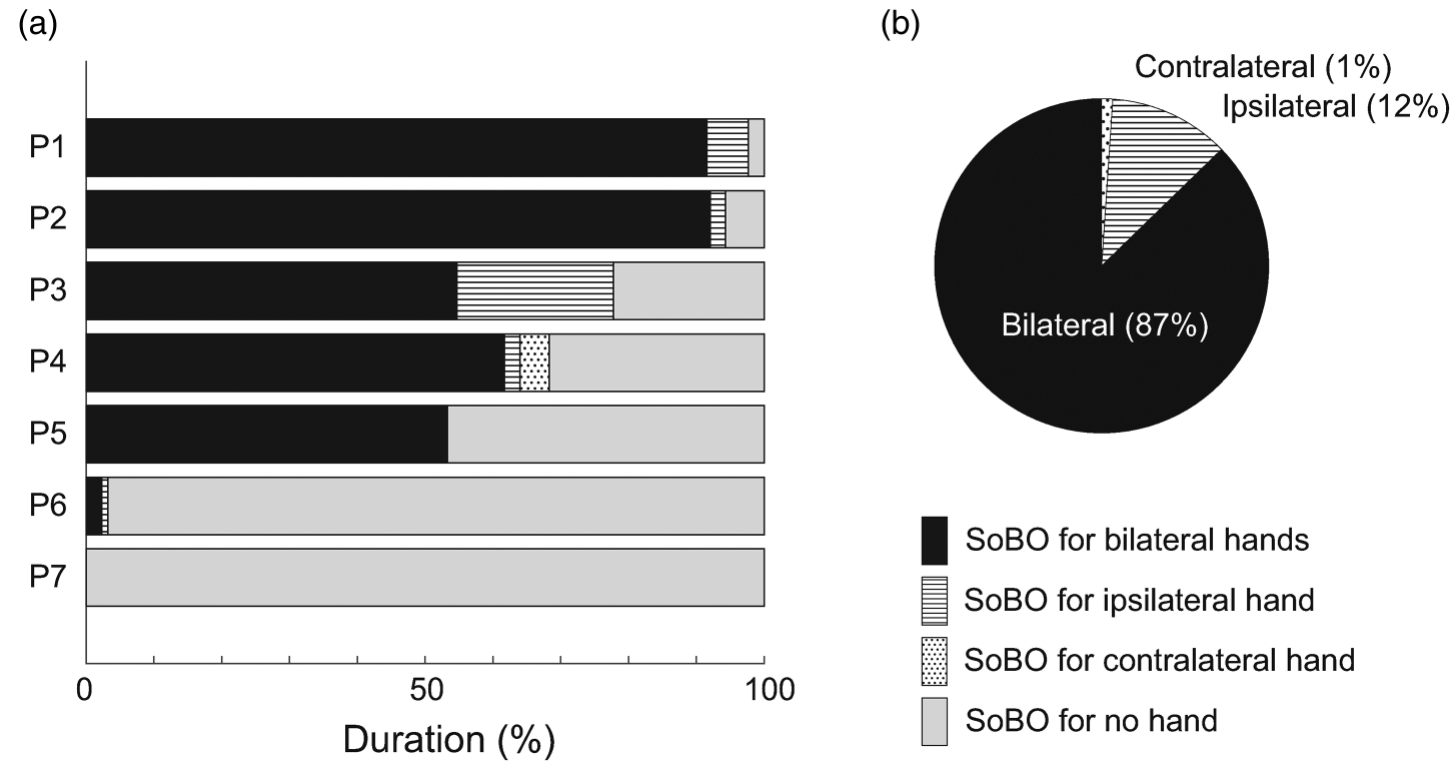
Bilateral rubber hand illusion. (a) The percentages of the duration in which the participants perceived the sense of body ownership (SoBO) for the bilateral hands, ipsilateral hand, contralateral hand, and no hand are shown for individual participants (P1–7). (b) The average percentages of the durations of three types of SoBO relative to that for any SoBO (calculated from the data of P1–6).

This study demonstrated that unilateral hand stimulation can induce bilateral hand embodiment when both fake hands are visible. This phenomenon termed the bilateral RHI indicates that the brain has an integrated representation of the SoBO in both hands. This suggests that concurrent visuo-tactile stimuli can induce not only partial ownership of a single body part (Botvinick & Cohen, 1998) or global ownership of the whole body (Ehrsson, 2007; Lenggenhager et al., 2007; Petkova et al., 2011) but also an intermediate type of ownership for multiple remote parts of the body, as far as they are visible. The present experimental protocol may contribute to addressing how these three types of SoBOs are represented in the brain (e.g., hierarchically or in parallel) and how multiple body parts are integrated into a unified SoBO or self-consciousness.

This study has some notable limitations and future directions. First, the representation of one hand interacts with that of the other hand during perception and motor action (Nozaki et al., 2006; Yamamoto & Kitazawa, 2001). Whether the SoBO for multiple remote parts of the body occurs only for both hands or for any combination of body parts (e.g., a hand and a leg) should be addressed. Second, we did not examine the extent of ownership in the present study. We also did not examine potential differences between subjective and implicit measures (e.g., Hansford et al., 2024). It would be interesting to examine the extent of SoBO for ipsilateral and contralateral hands by combining verbal reports and other implicit indices. Third, the sample size was small. Hence, rare phenomena, such as contralateral ownership, could not be evaluated adequately. Addressing these issues in future studies could contribute to understanding how the SoBO is shaped in the human brain.
